# Atrial Fibrillation Impacts Inpatient Mortality, Length of Stay, Resource Utilization, Blood Transfusion, and Endotracheal Intubation in Cesarean Sections, Natural Spontaneous Deliveries, and Instrumental Deliveries: A Nationwide Analysis (2016-2020)

**DOI:** 10.7759/cureus.74039

**Published:** 2024-11-19

**Authors:** Christian Siochi, Danny Segura Torres, Wilmer Cervantes, Marie Rabadi, Ricardo Machado Carvalhais, Peter Sobieraj, Stephen Jesmajian

**Affiliations:** 1 Internal Medicine, Montefiore New Rochelle Hospital, Albert Einstein College of Medicine, New Rochelle, USA

**Keywords:** atrial fibrillation, blood transfusion, cesarean section, endotracheal intubation, instrumental delivery, maternal morbidity, maternal mortality, normal spontaneous delivery, pregnancy, resource utilization

## Abstract

Background: Atrial fibrillation (AF) is rare during pregnancy and current data on the impact of AF during delivery is scarce. In this study, we aim to analyze the impact of AF in patients who underwent delivery via cesarean section (CS), natural spontaneous delivery (NSD), or instrumental delivery (ID).

Methods: This study analyzed discharge data from the National Inpatient Sample (NIS) from 2016 to 2020. Using the International Classification of Diseases, Tenth Revision, Clinical Modification (ICD-10-CM) and Procedure Coding System (ICD-10-PCS) codes, this study identified patients who underwent CS, NSD, or ID with a secondary diagnosis of AF. The study then compared these patients with patients who underwent CS, NSD, or ID without a secondary diagnosis of AF to analyze various outcomes. The primary outcome was all-cause in-hospital mortality. Secondary outcomes included length of stay, total hospital charges, blood transfusions, and respiratory failure requiring endotracheal intubation. STATA v.13 (StataCorp LLC, College Station, TX) was used for univariate and multivariate analysis.

Results: A total of 17,785,980 patients underwent CS, NSD, or ID. Of these deliveries, 6,000 patients had a secondary diagnosis of AF. Patients with AF had almost 20 times more of a chance of dying while admitted compared to those without AF (OR: 19.12; 95% CI: 4.33-84.45; p < 0.001). Furthermore, the AF cohort stayed for one and a half days longer in the hospital (regression coefficient: 1.55; 95% CI: 1.16-1.94; p < 0.001), spent 19,294.05 more dollars (regression coefficient: 19294.05; 95% CI: 14658.17-23929.93; p < 0.001), were subjected 2.68 times more to blood transfusions (OR: 2.68; 95% CI: 1.89-3.8; p < 0.001), and had a higher rate of respiratory complications requiring endotracheal intubation (OR: 15.86; 95% CI: 7.83-32.15; p < 0.001).

Conclusion: AF has a substantial negative impact on inpatient outcomes for pregnant patients during delivery. Further research is needed to explore these negative impacts to improve maternal care.

## Introduction

Atrial fibrillation (AF) is the most common cardiac arrhythmia in the United States and worldwide. The prevalence of AF in the United States was approximated at 5.2 million in the year 2010, with a projected increase to 12.1 million by the year 2030 [1]. With AF rising at exceedingly alarming rates, it is important to anticipate adverse outcomes of this growing epidemic.

Currently, there is a particular gap in the literature on the effects of AF on pregnancy delivery outcomes. Females exhibit a decreased incidence of AF compared to males; however, they demonstrate a higher propensity for experiencing negative outcomes associated with the condition, such as stroke, congestive heart failure, and mortality [2]. Sex differences exist in the initiation and progression of AF so it is imperative to explore the specific effects AF may have on pregnant women to improve care and management of this important population.

Pregnancy hospitalizations represent a significant population in the healthcare system. Between 2016 and 2020, discharge data from the National Inpatient Sample (NIS), Healthcare Cost and Utilization Project, Agency for Healthcare Research and Quality quantified 17,785,980 patients who underwent deliveries via cesarean section (CS), natural spontaneous delivery (NSD), or instrumental delivery (ID) [3]. Even without a past medical history of AF, pregnancy itself confers an inherent risk of morbidity and mortality. In the United States, the maternal mortality rate for 2020 was 23.8 deaths per 100,000 live births [4]. The trend of maternal mortality differs per reporting agency. One study reported that in-hospital maternal death rates decreased 57%, from 10.6 per 100,000 discharges in 2008 to 4.6 per 100,000 discharges in 2021, across the United States [5]. Opposingly, the National Vital Statistics System reports show that maternal mortality rates in the United States have nearly doubled, from 17.4 in 2018 to 32.9 per 100,000 live births in 2021 [6]. Regardless of the trend, these studies show that maternal mortality exists and there is a need to optimize maternal care.

While data on trends in maternal mortality may conflict, current literature does agree that maternal morbidity continues to rise. An analysis of NIS data conducted by Callaghan et al. reported that severe morbidity during delivery hospitalizations in 2008-2009 was recorded at 129 per 10,000 delivery hospitalizations [7]. Their NIS study found a notable increase of 75% in severe maternal morbidity for delivery hospitalizations when compared to data from a decade prior with escalated occurrences of blood transfusions, acute renal failure, shock, and respiratory distress syndrome. From 2008 to 2021, the prevalence of the same complications continued to rise, along with increased occurrence of mechanical ventilation and hysterectomy [5]. Combining these data, we observe a steady incline in maternal morbidity from 1998 to 2021.

Literature ranges regarding the contribution of AF to this observed increase in maternal morbidity. Some deem AF rare [8] with infrequent complications [9]. In an analysis involving 136,422 hospitalizations related to pregnancy from 1992 to 2000, only 0.1% of the cases were linked to arrhythmia with sinus arrhythmia being the most common diagnosis [8]. Their analysis found AF and atrial flutter to be rare with a diagnosis in 1% of cases. Another study noted infrequent complications from AF during pregnancy [9]. In 2016, Lee et al. found that among patients diagnosed with AF, maternal cardiac complications included only two cases of heart failure and no instances of stroke, systemic embolic events, or maternal mortality [9]. When they compared the AF cohort to individuals without AF, fetal birth weights did not differ significantly; however, the rate of neonatal intensive care unit (NICU) admissions was significantly higher among neonates born to mothers with AF (10.8% vs. 5.1%; P = 0.003). This increased rate of NICU admissions reveals that the impact of AF on pregnancy may not only affect maternal outcomes but it may affect fetal complications as well.

Other studies regarding the contribution of AF to the observed increase in maternal morbidity suggest that AF during pregnancy is increasing in prevalence with alarming outcomes [10-12]. In a larger nationwide study conducted by Vaidya et al. from 2000 to 2012, including over 57 million admissions of pregnant individuals, they estimated a rate of 68 sustained arrhythmias per 100,000 pregnancy-related hospitalizations [10]. Among these, AF was reported in 27 per 100,000 cases, and ventricular tachycardia (VT) in 16 per 100,000 cases. The frequency of arrhythmias, particularly AF and VT, rose throughout their study period and was linked to a higher occurrence of in-hospital mortality (5.9%) and maternal or fetal complications (36.5%) compared to admissions related to pregnancy without arrhythmias (0% and 21.8%, respectively) [10].

Since the data on the effects of AF on pregnancy are conflicting, further exploration is warranted to elucidate why and how AF affects pregnancy outcomes. The recent surge in AF and VT occurrences during pregnancy may be attributed to the increasing maternal age, along with the rising prevalence of risk factors like obesity, hypertension, and diabetes mellitus [11]. Congruent with the rise in risk factors, it is reasonable to assume that more pregnant women are entering their pregnancy with established comorbidities such as heart disease. A study that supports this notion showed that the prevalence of heart disease among pregnant women exhibited a notable rise of 24.7% between 2003 and 2012, primarily attributed to the surge in conditions such as cardiomyopathy, congenital heart disease, and pulmonary hypertension [12]. In this timeframe, the incidence of major adverse cardiac events also experienced a significant increase of 18.8% [12].

Although AF may be seemingly rare during pregnancy, cases are rising in number. Given the rise in maternal morbidity over the past two decades, and that the data on the impact of AF during delivery are scarce, further investigation is necessary due to the possible consequences of AF on pregnancy delivery outcomes.

Study aim

This study focuses on hospitalized patients who underwent delivery via CS, NSD, or ID with a secondary diagnosis of AF to analyze the impact of AF during delivery.

This article was previously presented as an abstract at the 2023 American Heart Association Scientific Sessions on November 13, 2023.

## Materials and methods

Study design

This was a retrospective cohort study using discharge data from the NIS, Healthcare Cost and Utilization Project (HCUP), Agency for Healthcare Research and Quality from 2016 to 2020.

Study inclusion criteria

Patients with a procedure code of cesarean section, natural spontaneous delivery, or instrumental delivery, aged >18 years, with or without a secondary diagnosis of AF were identified using the International Classification of Diseases, Tenth Revision, Procedure Coding System (ICD-10-PCS) and Clinical Modification (ICD-10-CM) codes.

Ethical considerations

The data from the NIS-HCUP are publicly available, deidentified, and exempt from institutional review board approval. The need for informed consent was waived.

Outcome measures

The primary outcome of interest was all-cause in-hospital mortality, while secondary outcomes included length of stay, total hospital charges, blood transfusions, and respiratory failure requiring endotracheal intubation.

Statistical analysis

This study used a confidence interval (CI) of 95% and a two-tailed p-value < 0.05 as statistically significant in its analysis. Continuous variables were examined through the calculation of means accompanied by standard deviations or medians along with interquartile ranges in the case of normally distributed and skewed data, respectively. Descriptive statistics incorporating frequencies and percentages were employed for the analysis of categorical variables. Patient and hospital-level baseline characteristics, and in-hospital outcomes were compared between patients with a procedure code of cesarean section, natural spontaneous delivery, or instrumental delivery, aged > 18 years, with or without a secondary diagnosis of AF using the Pearson χ2 test for categorical variables and the independent sample t-test for continuous variables. To calculate unadjusted and adjusted odds ratios for in-hospital clinical outcomes, univariate and multivariate logistic regression were used. To account for potential confounding factors, a univariate analysis identified patient and hospital-level baseline characteristics with a p-value < 0.2. These variables were then adjusted in a multivariate regression model. All analyses were conducted using STATA v.13 (StataCorp LLC, College Station, TX).

## Results

Baseline patient characteristics, including race and Charlson Comorbidity Index, and hospital-level characteristics are shown in Table 1. A total of 17,785,980 patients, aged >18 years with a procedure code of CS, NSD, or ID in the United States between 2016 and 2020 were included in the final cohort. In the final cohort, 6,000 patients had a secondary diagnosis of AF while 17,779,980 patients did not have a secondary diagnosis of AF. The AF population had a mean age of 31.23 years, while those without AF had a mean age of 29.13 years.

**Table 1:** Percentage of patients with a procedure code of cesarean section, natural spontaneous delivery, or instrumental delivery, per baseline patient and hospital-level characteristics, with or without a secondary diagnosis of atrial fibrillation. To account for potential confounding factors, a multivariate regression model was later adjusted for patient and hospital-level baseline characteristics with a p-value < 0.2. AF: atrial fibrillation.

Baseline characteristics	% without AF (N = 17,779,980)	% with AF (N = 6,000)	% total (N = 17,785,980)	p-value
Race				
White	52.76	51.07	52.75	<0.01
Black	14.97	30.52	14.97	
Hispanic	20.71	10.4	20.71	
Asian	6.28	3.84	6.28	
Native American	0.72	0.85	0.72	
Other	4.56	3.32	4.56	
Charlson Comorbidity Index				
0	92.91	75	92.9	<0.01
1	6.65	18.25	6.65	
2	0.36	4.42	0.36	
>3	0.0008	2.33	0.009	
Median income				
<49999	27.66	32.26	27.66	0.005
50000-64999	25.37	24.47	25.37	
65000-85.999	24.78	23.12	24.78	
>86000	22.2	20.15	22.2	
Insurance type				
Medicare	0.72	3.44	0.72	<0.01
Medicaid	43.14	43.56	43.14	
Private insurance	53.54	51.26	53.54	
Self-pay	2.6	1.72	2.6	
Hospital region				
Northeast	15.95	16.33	15.95	0.04
Midwest	21.09	21.75	21.09	
South	39.2	41.83	3.92	
West	23.76	20.08	23.76	
Hospital bed size				
Small	19.28	13.67	19.27	<0.01
Medium	30.18	23.75	30.17	
Large	50.55	62.58	50.55	
Hospital location				
Rural	8.99	5.96	8.99	<0.01
Urban	91.01	94.04	91.01	
Hospital teaching status				
No	29.94	19.33	29.94	<0.01
Yes	70.06	80.67	70.06	

The majority of patients with and without AF were of White ethnicity, 51.07% (N = 3,064) and 52.76% (N = 9,380,717), respectively. The most common Charlson Comorbidity Index score was 0 in both the AF and no AF groups. A detailed description of the baseline demographics, Charlson Comorbidity Index, and hospital-level characteristics is provided in Table 1.

Primary outcome

Patients with AF had almost 20 times more of a chance of all-cause in-hospital mortality compared to those without AF (OR: 19.12; 95% CI: 4.33-84.45; p < 0.001) (Table 2).

**Table 2:** The adjusted odds ratio for mortality outcome of patients with a procedure code of cesarean section, natural spontaneous delivery, or instrumental delivery, with a secondary diagnosis of AF compared to those without AF. This study used a confidence interval of 95% and a p-value < 0.05 as statistically significant in its analysis. AF: atrial fibrillation.

Primary outcome adjusted for variables with p < 0.2							
In-patient mortality	Odds ratio	Std. error	t	p-value	95% confidence interval		Total outcome (n)/total outcome with AF (%)
Atrial fibrillation/total deliveries	19.12	14.49	3.89	<0.001	4.33	84.45	1130/0.25%
Atrial fibrillation/cesarean section	13.76	9.98	3.62	<0.001	3.32	57.03	935/0.31%
Atrial fibrillation/natural delivery	63.96	89.35	2.98	0.003	4.14	988.87	165/0.2%
Atrial fibrillation/instrumental delivery	-	-	-	-	-	-	35/0%

Secondary outcomes

The AF cohort stayed for one and a half days longer (regression coefficient: 1.55; 95% CI: 1.16-1.94; p < 0.001) and spent 19,294.05 more dollars during their hospital stay (regression coefficient: 19294.05; 95% CI: 14658.17-23929.93; p < 0.001) (Table 3).

**Table 3:** The adjusted regression coefficient for the length of hospital stay and total hospital charges outcomes of patients with a procedure code of cesarean section, natural spontaneous delivery, or instrumental delivery, with a secondary diagnosis of AF compared to those without AF. This study used a confidence interval of 95% and a p-value < 0.05 as statistically significant in its analysis. AF: atrial fibrillation.

Secondary outcomes adjusted for variables with p < 0.2							
Length of stay	Coefficient	Std. error	t	p-value	95% confidence interval		Mean outcome/mean outcome with AF
Atrial fibrillation/total deliveries	1.55	0.19	7.78	<0.001	1.16	1.94	2.63/4.41
Atrial fibrillation/cesarean section	1.64	0.33	5	<0.001	1	2.28	3.43/5.36
Atrial fibrillation/natural delivery	0.79	14.17	5.55	<0.001	0.51	1.07	2.23/3.14
Atrial fibrillation/instrumental delivery	3.28	1.27	2.58	0.01	0.79	5.77	2.48/5.44
Total hospital charges	Coefficient	Std. error	t	p-value	95% confidence interval		Mean outcome/mean outcome with AF
Atrial fibrillation/total deliveries	19294.05	2365.13	8.16	<0.001	14658.17	23929.93	21357.24/42881.02
Atrial fibrillation/cesarean section	23139.42	4044.14	5.72	<0.001	15212.55	31066.29	30299.61/55838.27
Atrial fibrillation/natural delivery	8715.58	1412.35	6.17	<0.001	5947.24	11483.91	16912.1/26527.13
Atrial fibrillation/instrumental delivery	37919.06	12018.34	3.16	0.002	14362.07	61476.06	19637.23/52449.06

The AF cohort was subjected 2.68 times more to blood transfusions (OR: 2.68; 95% CI: 1.89-3.8; p < 0.001) and had a higher rate of respiratory complications requiring endotracheal intubation during their hospital stay (OR: 15.86; 95% CI: 7.83-32.15; p < 0.001) (Table 4).

**Table 4:** The adjusted odds ratio for the length of hospital stay and total hospital charges outcomes of patients with a procedure code of cesarean section, natural spontaneous delivery, or instrumental delivery, with a secondary diagnosis of AF compared to those without AF. This study used a confidence interval of 95% and a p-value < 0.05 as statistically significant in its analysis. AF: atrial fibrillation.

Additional secondary outcomes adjusted for variables with p < 0.2							
Endotracheal intubation	Odds ratio	Std. error	t	p-value	95% confidence interval		Total outcome (n)/total outcome with AF (%)
Atrial fibrillation/total deliveries	15.86	5.72	7.67	<0.001	7.83	32.15	6555/1.25%
Atrial fibrillation/cesarean section	12.31	4.49	6.89	<0.001	6.02	25.16	5315/2.03%
Atrial fibrillation/natural delivery	6.64	11.6	1.08	0.28	0.22	203.85	1015/0.2%
Atrial fibrillation/instrumental delivery	74.41	65.96	4.86	<0.001	13.09	422.91	300/3.23%
Blood transfusion	Odds ratio	Std. error	t	p-value	95% confidence interval		Total outcome (n)/total outcome with AF (%)
Atrial fibrillation/total deliveries	2.68	0.48	5.54	<0.001	1.89	3.8	192635/3.75%
Atrial fibrillation/cesarean section	2.04	0.44	3.32	0.001	1.34	3.1	118600/0.53%
Atrial fibrillation/natural delivery	2.85	1.07	2.81	0.005	1.37	5.93	65520/1.78%
Atrial fibrillation/instrumental delivery	6.74	3.21	4	<0.001	2.65	17.17	11140/8.06%

## Discussion

The results of this study support that AF has a substantial negative impact on inpatient outcomes for pregnant patients during delivery. Pregnant patients with AF who underwent delivery via CS, NSD, or ID had a statistically significant higher chance of all-cause in-patient mortality, longer hospital stays, more resource utilization, blood transfusion, and respiratory complications requiring intubation.

The primary outcome of interest in this investigation was all-cause in-patient mortality during pregnancy hospitalization. One plausible explanation for the increase in mortality among pregnant patients with AF is that the physiologic hypercoagulability in normal pregnancy compounded by the rapid, irregular heartbeats of AF further increases the risk of adverse thrombotic events. A typical pregnancy brings about significant modifications in the coagulation and fibrinolytic mechanisms [13]. These inherent procoagulant adjustments serve the purpose of reducing blood loss during childbirth, yet they also elevate the likelihood of thromboembolism in both antenatal and postpartum phases [13]. These alterations lead to a hypercoagulable state during pregnancy, which may persist for a minimum of eight weeks after childbirth [14]. This results in a three to four times increase in arterial thromboembolism (such as strokes and myocardial infarction) and a four to five times increase in venous thromboembolism during pregnancy; moreover, there is a substantially heightened risk (20 times greater) in the postpartum period compared to non-pregnant females [14]. As normal physiologic pregnancy already confers a risk of adverse events, pathologies during pregnancy, such as AF, would further increase the already elevated risk.

Obstetric complications may also lead to the development of arrhythmias [7]. In 2016, Lee et al. posited that while the precise mechanism elucidating the connection between obstetric complications and AF remains unknown, it is possible that obstetric complications, such as preeclampsia, may result in an increased adrenergic state, heightened inflammatory response, as well as activation of the renin‐angiotensin‐aldosterone system [7]. Their study noted that these factors could potentially trigger electrophysiological changes in the atrium and elevate susceptibility to arrhythmias.

Obstetric complications such as preeclampsia may also increase the risk of severe postpartum hemorrhage (PPH) necessitating blood transfusion [15]. A study done in 2013 examined a total of 8,571,209 deliveries, out of which 25,906 (equivalent to 3.0 per 1000) were complicated by PPH [15]. The definition of severe PPH in their investigation encompassed PPH alongside the administration of a blood transfusion, hysterectomy, and/or surgical repair of the uterus. Noteworthy risk factors that emerged included preeclampsia (adjusted odds ratio (aOR): 3.1; 95% CI: 2.9-3.3) [15].

However, while efforts have been made to recognize patients with a heightened susceptibility to PPH, some studies suggest that this critical complication frequently manifests in women devoid of discernible risk factors [16]. Since data regarding risk factors leading to PPH are conflicting, additional investigation is imperative to delineate a definitive relationship among preeclampsia, AF, and severe PPH necessitating blood transfusions.

Additionally, although blood transfusion is frequently indispensable in obstetrics, the utilization of blood components, especially during massive transfusions, is accompanied by inherent risks. These risks include transfusion-related acute lung injury, acute respiratory distress syndrome, and transfusion-associated circulatory overload [16]. This transfusion-related respiratory sequelae may complicate a delivery hospitalization, possibly resulting in the need for intubation.

Another explanation for the increased mortality in delivery hospitalizations could be attributed to the increased prevalence of established cardiovascular disease in the target population. The higher prevalence of cardiovascular disease is furthermore correlated with a heightened susceptibility to arrhythmias as well as cardiac arrest [17]. An analysis of 2.2 million hospital admissions related to pregnancy revealed that cardiac arrest and arrhythmias were more prevalent in expectant mothers with cardiovascular disease compared to those lacking cardiovascular disease (0.3% and 7.2% versus 0.004% and 0.3%, respectively) [17].

As an expectant mother’s past medical history gets more complicated, delivery outcomes can be expected to worsen, explaining this study’s findings of an increase in resource utilization. Fink et al. compared the frequency of maternal co-morbid conditions from 2008 to 2021 and found an increased occurrence of gestational hypertension, severe preeclampsia, pre-existing hypertension, substance use disorder, asthma, gestational diabetes, obesity, and hemorrhage [5]. Their study supports that severe maternal morbidities are known risk factors for maternal deaths and impose substantial social and economic burdens.

Additionally, as an expectant mother’s age becomes more advanced, delivery outcomes can be expected to worsen [5,18,19]. A study conducted in the United States spanning from 2006 to 2015 revealed a rise of 4% in childbirths among 25-29-year-old patients, 18% in 30-34-year-old patients, 5% in 35-39-year-old patients, 8% in 40-44-year-old patients, and 26% among 45-54-year-old patients [18]. Advanced maternal age may increase the risk of developing arrhythmias [11]. Thus, the increase in pregnancies among those with advanced maternal age may have contributed to the rising number of pregnant patients with AF. The increasing prevalence of pregnancies complicated by AF likely contributed to the steady incline in maternal morbidity observed in the past two decades, offering an explanation for this study’s findings of an increase in mortality among pregnant patients with AF.

Limitations

The NIS serves as a repository of hospital administrative data, lacking the depth of clinical information found in electronic health records. The identification of clinical conditions and procedures is dependent on the precision of diagnosis and procedure codes reported by hospitals. Inaccuracies in coding have the potential to result in the misclassification of data variables.

Due to limitations in accessible data, this study could not differentiate between chronic versus new-onset AF, though temporality may differentially affect outcomes in pregnancy. A past medical history of arrhythmias is a significant determinant of the likelihood of experiencing repeated episodes of arrhythmias while pregnant [20]. It is important to note that new-onset, “lone” AF may also occur with seemingly less deleterious effects. One case report noted a pregnant patient with no comorbidities, presenting with new-onset “lone” AF without hemodynamic compromise [21]. Their patient responded well to intravenous diltiazem; the AF episode resolved in less than 48 hours, so the patient was discharged with no anticoagulation. Another case report noted a 40-year-old with a cerclage due to an incompetent cervix and previous preterm deliveries, presenting with new-onset “lone” AF [22]. Their patient was treated with diltiazem as well, with a short course of digoxin, and subsequently converted to sinus rhythm. The lack of differentiation in this investigation between chronic and new-onset AF could be a limitation in the interpretation of results.

The definition of important data variables in this study such as comorbidities and mortality should be addressed. Maternal comorbid conditions were quantified using the Charlson Comorbidity Index, which may lead to an incomplete representation of the target population’s pre-existing conditions. For example, the Charlson Comorbidity Index inquires about a history of congestive heart failure and myocardial infarction but not congenital heart defects, which is a commonly known cause of AF. Additionally, the definition of mortality was based on in-hospital death during the visit for delivery, without accounting for death before delivery admission or after discharge.

This study was a retrospective observational investigation, thus encompassing all inherent constraints linked with such analyses. The allocation of treatments among various groups lacked randomization, posing a challenge in ascertaining the specific factors contributing to certain outcomes, such as maternal arrhythmias or the impact of the pharmacological interventions prescribed for arrhythmia management. The database did not contain information regarding exposure to medications, making it infeasible to assess medication adherence for individual patients. Additional prospective studies should be conducted to address these limitations and further enhance the depth of knowledge on why and how AF affects maternal delivery outcomes.

Notwithstanding the limitations mentioned above, the extensive size of the sample in this research undeniably enhances the statistical significance of its results.

## Conclusions

The concurrent escalation of AF prevalence and maternal morbidity in the United States, both phenomena influenced in part by the trend of advancing maternal age and increasing presence of comorbidities, prompts an understanding of the effects of AF on pregnancy outcomes. This investigation revealed that AF has a substantial negative impact on pregnant patients during delivery. Pregnant patients with AF who underwent delivery via CS, NSD, or ID had a statistically significant higher chance of all-cause in-patient mortality, longer hospital stay, more resource utilization, blood transfusion, and respiratory complications requiring intubation. These findings underscore the importance of additional investigation regarding the etiology of mortality and severe maternal morbidity, encompassing the influence of comorbidities such as AF on maternal and fetal outcomes. Further studies on the impact of AF on pregnancy and delivery can optimize multidisciplinary and integrative strategies for maternal care to reduce the rates of maternal morbidity and mortality.
